# Changing knowledge, attitudes and behaviours towards cytomegalovirus in pregnancy through film-based antenatal education: a feasibility randomised controlled trial of a digital educational intervention

**DOI:** 10.1186/s12884-021-03979-z

**Published:** 2021-08-18

**Authors:** Anna Calvert, Tushna Vandrevala, Robin Parsons, Victoria Barber, Alex Book, Gayle Book, David Carrington, Vanessa Greening, Paul Griffiths, Danielle Hake, Asma Khalil, Suzanne Luck, Amy Montague, Caroline Star, Irina Chis Ster, Sharon Wood, Paul T. Heath, Christine E. Jones

**Affiliations:** 1 Paediatric Infectious Diseases Research Group, St George’s, University of London, Cranmer Terrace, London, SW17 0RE UK; 2 St George’s University Hospitals NHS Foundation Trust, London, UK; 3 Department of Psychology, Kingston University, Kingston-Upon-Thames, UK; 4 Parent Caring for a Child With Congenital CMV Infection, London, UK; 5 University College London, Medical School, Institute of Immunity and Transplantation, London, UK; 6 Kingston Hospital NHS Foundation Trust, Kingston-Upon-Thames, UK; 7 CMV Action, London, UK; 8 Institute of Infection and Immunity, St George’s University of London, London, UK; 9 Faculty of Medicine and Institute for Life Sciences, University of Southampton, and NIHR Southampton Clinical Research Facility and NIHR Southampton Biomedical Research Centre, University Hospital Southampton NHS Foundation Trust, Southampton, UK

**Keywords:** Cytomegalovirus, Congenital infection, Pregnancy, Antenatal education, Feasibility

## Abstract

**Background:**

Congenital cytomegalovirus (CMV) is the most common congenital infection globally, however information about CMV is not routinely included in antenatal education in the United Kingdom. This feasibility study aimed to gather the essential data needed to design and power a large randomised controlled trial (RCT) to investigate the efficacy of a digital intervention in reducing the risk of CMV acquisition in pregnancy. In order to do this, we carried out a single-centre RCT, which explored the knowledge, attitudes and risk reduction behaviours in women in the intervention and treatment as usual groups, pre- and post-intervention.

**Methods:**

CMV seronegative women living with a child less than four years old, receiving antenatal care at a single UK tertiary centre, were randomised to the digital intervention or ‘treatment as usual’ groups. Participants completed questionnaires before the digital intervention and after and at 34 gestational weeks, and responses within groups and between groups were compared using tailored randomisation tests. CMV serology was tested in the first trimester and at the end of pregnancy.

**Results:**

Of the 878 women screened, 865 samples were analysed with 43% (*n* = 372) being CMV seronegative and therefore eligible to take part in the RCT; of these, 103 (27.7%) women were enrolled and 87 (84%) of these completed the study. Most participants (*n* = 66; 64%) were unfamiliar with CMV at enrolment, however at 34 gestational weeks, women in the intervention group (*n* = 51) were more knowledgeable about CMV compared to the treatment as usual group (*n* = 52) and reported engaging in activities that may increase the risk of CMV transmission less frequently. The digital intervention was highly acceptable to pregnant women. Overall, four participants seroconverted over the course of the study: two from each study group.

**Conclusions:**

A large multi-centre RCT investigating the efficacy of a CMV digital intervention is feasible in the United Kingdom; this study has generated essential data upon which to power such a study. This single-centre feasibility RCT demonstrates that a digital educational intervention is associated with increase in knowledge about CMV and can result in behaviour change which may reduce the risk of CMV acquisition in pregnancy.

**Trial registration:**

Clinicaltrials.gov, NCT03511274, Registered 27.04.18, http://www.Clinicaltrials.gov

**Supplementary Information:**

The online version contains supplementary material available at 10.1186/s12884-021-03979-z.

## Background

Congenital cytomegalovirus (CMV) is the commonest congenital infection globally and has a birth prevalence of 0.3–1% [1–3]. Congenital CMV (cCMV) can occur following the first infection with CMV during pregnancy (primary CMV infection), after reactivation of CMV acquired previously or following infection with a different strain of CMV (secondary CMV infection). The risk of transmission to the fetus is significantly higher in primary infection than in secondary infection [4]. Despite this, globally more infants with cCMV are born to mothers with secondary infection than with primary infection due to the high CMV seroprevalence in many parts of the world [4]. CMV is transmitted through contact with infected bodily fluids and those people who have a child, or children, already are at increased risk of acquiring the infection, primarily through contact with infected saliva or urine from their young child [5].

The clinical spectrum of cCMV at birth is wide: around 85% of infants will be ‘asymptomatic’ and 15% will have symptoms at birth [6]. Long term sequelae occur in about 40–60% of babies who are symptomatic at birth, and 10–15% of babies who are asymptomatic [2]. The most common long-term effect of cCMV is sensorineural hearing loss, with cCMV being the most frequent non-genetic cause of sensorineural hearing loss and the only preventable cause [7]. cCMV represents a significant public health problem, but there are currently no licensed vaccines and no routinely recommended treatments for antenatal CMV infection. The United Kingdom (UK) currently has no national screening programme for CMV for pregnant women or infants, [8] and women are not routinely counselled about CMV risk reduction measures.

Antenatal education about CMV risk reduction may provide a significant opportunity to reduce CMV infection in pregnancy and consequently reduce the incidence of cCMV. In a recent systematic review, seven studies were identified which investigated preventative hygiene-based interventions in pregnancy or in women of child-bearing age [9]. This concluded that hygiene-based interventions in pregnancy could play a useful role in primary prevention of CMV infection in pregnancy, however the studies were too heterogeneous in terms of study population, intervention and outcome to form firm conclusions on the relative impact of such interventions. Additionally, the majority of interventions would not be easily translatable to routine antenatal care, without the provision of significant additional resources.

A randomised controlled trial (RCT) using an acceptable educational intervention—which can be subsequently integrated into routine care in the UK—is urgently needed.

RACE FIT (Reducing Acquisition of CMV through antenatal Education) was designed to inform the feasibility and design of a large-scale RCT in a UK setting to investigate the efficacy of the educational intervention on the risk of acquiring CMV infection in pregnancy. It was designed in two phases, the first of these involved in-depth interviews with pregnant women and the families of children affected by cCMV. These interviews explored their knowledge and attitudes about CMV, and perspectives on infection prevention in pregnancy, in order to prioritise themes to include in the intervention [10, 11]. From these findings, a script was produced and the digital intervention developed as a short educational film through an iterative process involving review by pregnant or recently pregnant women, families affected by CMV, and knowledge experts. The aim of this second phase of RACE FIT was to test the digital intervention in a feasibility study where women were randomised to the intervention or treatment as usual groups, in order to provide information about recruitment and conduct of a future trial, assess the acceptability of the educational intervention and explore changes in knowledge, attitudes and behaviours in the two groups. We also determined CMV seroconversion in both groups. The overarching aim was to inform the feasibility and design of a large-scale randomised controlled trial (RCT) in a UK setting to investigate the efficacy of the digital, antenatal educational intervention on the risk of acquiring CMV infection in pregnancy.

## Methods

### Study setting and screening

We recruited all participants from a single teaching hospital in an ethnically diverse area of South-West London. We approached women in their first trimester of pregnancy who were attending antenatal clinics between September 2018 and September 2019; women who lived with a child or children less than four years of age were asked for their consent for CMV serology to be undertaken on an additional blood sample. All women in the study were tested for both CMV IgG and IgM antibodies. Women who were seronegative (no evidence of previous CMV infection; IgG negative) were invited to take part in the RCT; those who were CMV IgG positive and CMV IgM negative were not eligible to take part and those who were CMV IgM positive had additional serology undertaken including CMV IgG avidity testing. Women with serological evidence of recent CMV infection were referred for counselling and further investigation under an established routine clinical pathway.

### Eligibility

Women were considered to be eligible for the RCT if they were aged over 18, pregnant, willing and able to provide informed consent, seronegative for CMV, having no documented immunodeficiency, living with at least one child aged less than four and willing to be followed up until delivery.

### Randomisation

After providing informed consent, participants were randomised in a 1:1 ratio to the intervention or treatment as usual group using the randomisation service provided by the King’s College Clinical Trials Unit. The randomisation sequence was computer generated. Neither the participant nor the researcher was blinded to group allocation.

### Study materials

Participants who were randomised to the intervention group watched the educational film—developed in phase one—at their first study visit. The film was made up of three parts: a presentation of facts about CMV, including prevalence and routes of transmission; families of affected children telling their stories; and advice provided about how the risk of infection could be reduced (Supplementary material 1). Participants in the treatment as usual group viewed a series of slides about influenza vaccination in pregnancy. Influenza vaccination is routinely recommended in pregnancy in the UK and all pregnant women receive information about this as part of routine care.

### Study design

The study was approved by the NHS Health Research Authority and South-Central Oxford Research Ethics Committee (16/SC/0683).

Women had their first study visit at home or in clinic before 16 gestational weeks. Following informed written consent, all participants completed a questionnaire (Supplementary material 2) and were then randomised into either the intervention or treatment as usual groups. Participants then either watched the digital educational intervention (intervention group) or reviewed a series of slides about influenza vaccination in pregnancy (treatment as usual group) and then immediately completed a second questionnaire about the materials they had been presented with (Supplementary material 3 and 4). At 34 gestational weeks, participants completed a final online questionnaire (Supplementary material 5 and 6). Within two weeks of delivery a blood sample was obtained from all participating mothers. This was tested for CMV specific IgG and IgM antibody to assess for seroconversion over the study period. Clinical follow up was organised for those participants and their infants who were found to have seroconverted since initial screening.

### Measures.

#### Participant demographics

Information was collected about age, marital status, ethnicity, length of residence in the UK, qualifications, number of previous pregnancies, number of children under four years of age and whether participants worked regularly with children as part of their job.

#### Familiarity with CMV

At baseline, participants indicated how familiar they were with a range of conditions affecting newborns, including CMV, and about how common they thought these conditions were [12].

#### Response to materials

At the first study visit, the intervention group provided their responses to the educational film by indicating their level of agreement with a range of statements.

For the following domains, participants in both groups were asked for their responses at baseline and at 34 weeks:

#### Knowledge of CMV:

Participants were asked to specify their level of agreement with 12 statements about CMV [12]. These included both true and false statements.

#### Perceived severity and susceptibility

Participants were asked to indicate their level of agreement with statements about the severity of CMV and their perceived susceptibility to CMV.

#### Anxiety and depression scores

Participants were asked to indicate how they had been feeling recently using the Kessler Psychological Distress Scale [13] and the Edinburgh Postnatal Depression Scale [14].

#### Daily activities

Participants were asked how often they engaged in a range of behaviours [12] relating to contact with a child’s saliva, urine or faeces. At 34 gestational weeks, participants were asked to indicate how hard it had been to make the suggested behavioural changes.

### Laboratory methods

CMV IgM and IgG were measured using the Roche Elecsys assay (Roche, Switzerland), according to manufacturer’s instructions. For individuals who were found to be CMV IgM positive further testing was performed for IgG avidity using the VIDAS CMV IgG avidity 11 assay (Biomerieux, France).

### Data collection and analysis

Study data were collected and managed using REDCap electronic data capture tools hosted at St George’s, University of London.

### Statistical analyses

Data were graphically explored and summarised. Anxiety and depression scores, which exhibited a wide range of values (additive scores), were treated as continuous data. Outcomes reflecting measurements for familiarity, attitudes, behaviour and knowledge were of ordinal type. Missing responses were assessed for each variable of interest. Both per-protocol (PP) and intention-to-treat (ITT) analyses were conducted [15]. Given the randomisation, permutation tests have been conducted for between groups comparisons assuming that the missing observations were completely at random [15–19]. Similar assumptions were considered for within groups’ comparisons. The PP and ITT analyses did not show markable qualitative differences for any of the outcomes.

This study aimed to detect potentially important signals to be investigated in a larger trial and was not designed as a hypotheses testing study. Given the exploratory phase of this research, classical Bonferroni corrections for multiple outcome testing were not applied.

All analyses and graphics have been produced using STATA 16 (StataCorp. 2019. Stata Statistical Software: Release 16. College Station, TX: StataCorp LLC).

## Results

### Screening for participation

**Fig. 1:**
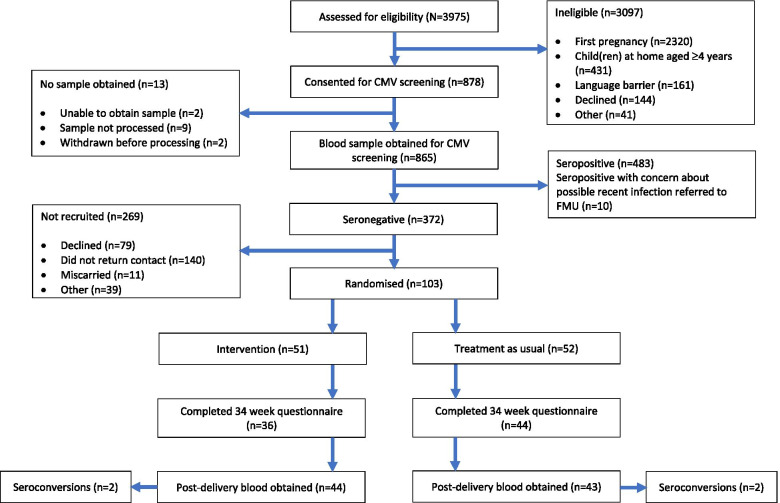
CONSORT flow diagram. CMV Cytomegalovirus; FMU Fetal Medicine Unit

A large number of women were approached about the study (*n* = 3975), of whom 878 (22%) had a blood sample taken for CMV serology, Fig. 1.

The most common reason for ineligibility for blood sampling was not living with a child aged less than four (*n* = 2751; 88.8%).

Overall, 43% (*n* = 372) of participants were seronegative and eligible to be approached about participation in the RCT and 57% (*n* = 493) of women were seropositive. Of all the women screened, ten (1.16%) had evidence indicating recent infection, within the last three months, and were referred to the Fetal Medicine Unit for further clinical investigation. Details of ethnicity were available for 532 women screened. The proportion of women who were seronegative varied by self-defined ethnicity: 61% White British (*n* = 172), 39% White Other (*n* = 32), 6% Black (*n* = 3), 22% South Asian (*n* = 21), 14% Asian Other (*n* = 2), 46% Mixed (*n* = 6).

### Feasibility randomized controlled trial

Of the 372 women who were CMV seronegative, 103 women consented to participate in the RCT (27.7%), of whom 87 (84%) participants completed the study (Fig. 1). Study completion was defined as collection of a final blood sample or completion of a 34-week questionnaire. Recruitment ended at the conclusion of the pre-defined recruitment period of 12 months. At that time, we had recruited about 25% of the initially planned recruitment number.

### Participant characteristics

The demographic characteristics of the participants are shown in Table 1.

**Table 1:**
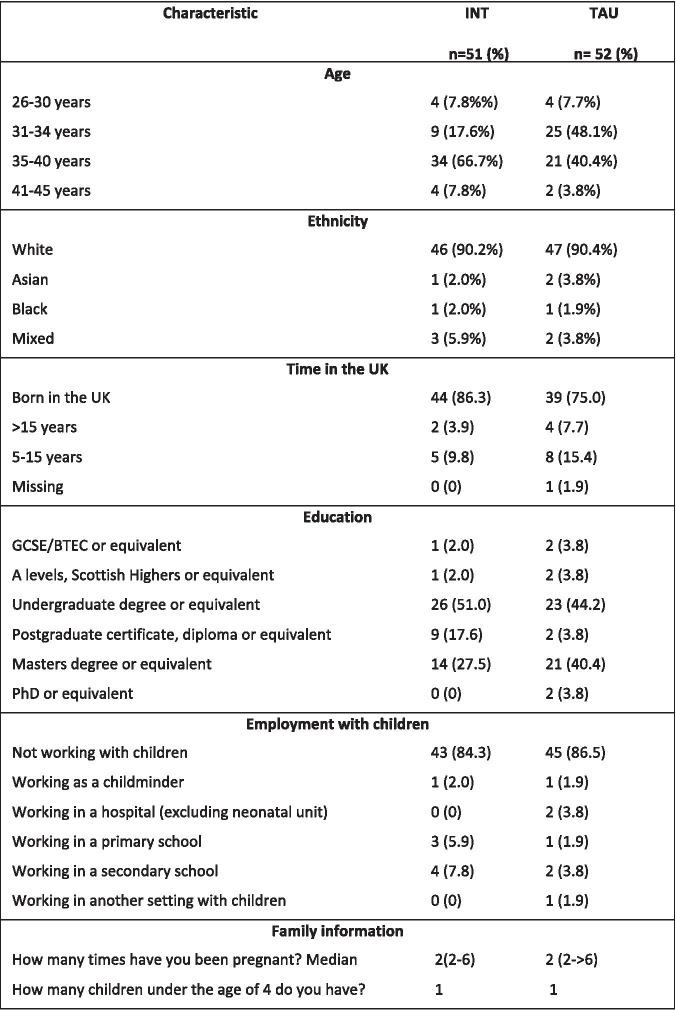
Demographic characteristics of participants

*INT* Intervention group; *TAU *Treatment As Usual group

### Familiarity with CMV and other conditions

On enrolment to the study, most participants who responded were unfamiliar with CMV; 64% (*n* = 66) of participants reported that they were ‘not at all familiar’ with CMV compared with 1% (*n* = 1) for Trisomy 21, 9% for rubella (*n* = 9), 13% (*n* = 13) for listeria and 32% (*n* = 32) for toxoplasmosis. There was no evidence to suggest any difference between the distribution of the responses in the two randomisation groups (Supplementary material 7; Fig. 1).

### Participants' knowledge about CMV

Knowledge about how CMV is transmitted and what effect congenital CMV can have on infants was consistent between randomisation groups, with no differences at baseline. At 34 gestational weeks, knowledge about CMV was significantly different between participants in the intervention group and participants in the treatment as usual group; a higher proportion of participants in the intervention group correctly agreed that CMV can be spread through saliva and urine, and could cause hearing loss and adverse neurodevelopmental outcomes, Table 2. Within the intervention group, there was a significant difference in knowledge about transmission of CMV and the potential consequences of congenital CMV for the infant or child, at baseline compared to 34 gestational weeks, Table 2. Knowledge about how CMV can be transmitted was also different at 34 gestational weeks compared to baseline in the treatment as usual group, however there was not a significant difference in knowledge about the impact of CMV on hearing and development in this group, suggesting the participants gained some knowledge about CMV during the study period, despite not being exposed to the intervention, Table 2.

**Table 2:**
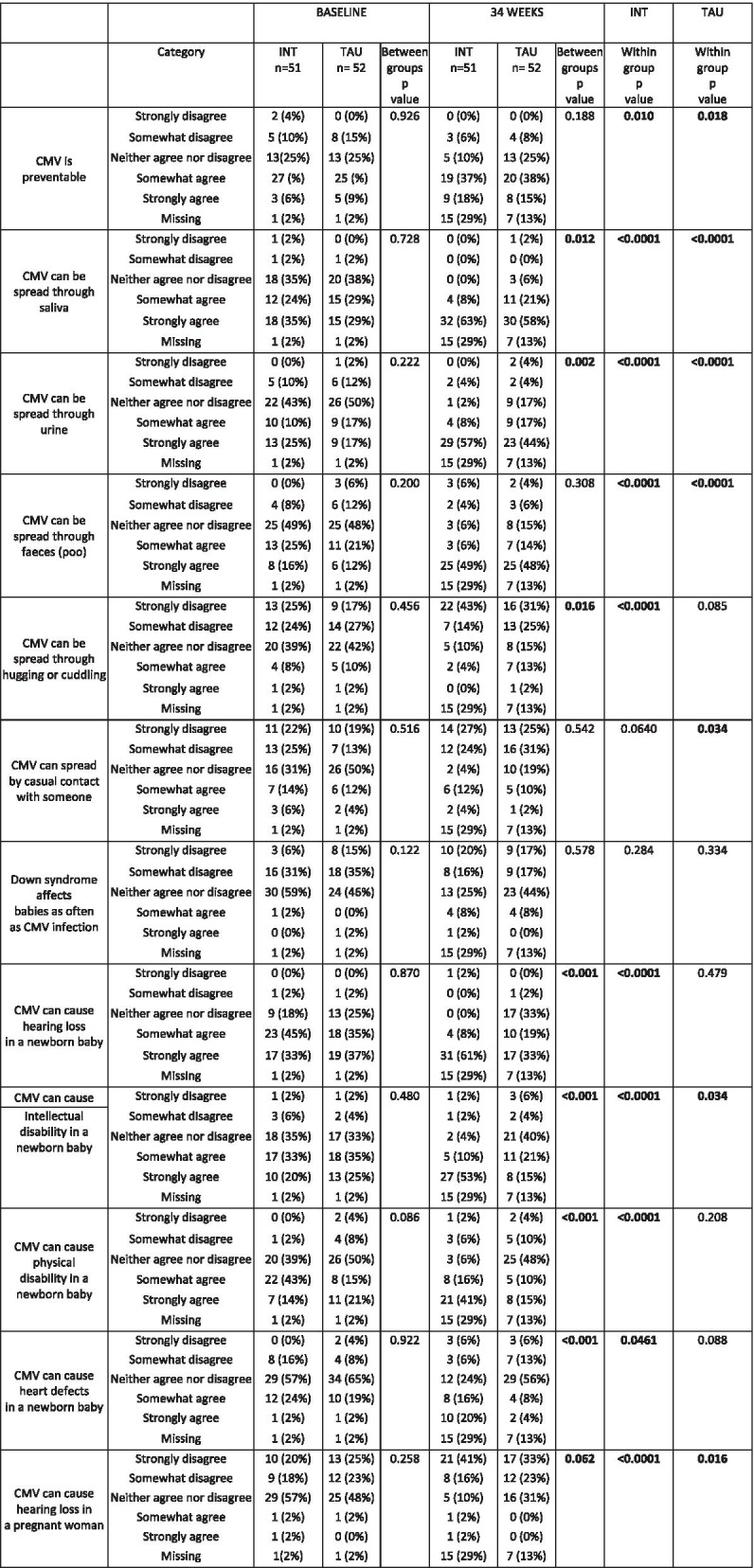
Knowledge of CMV in participants at baseline and 34 weeks

*CMV* Cytomegalovirus; *INT* Intervention group; *TAU* Treatment As Usual group

### Perception of severity and susceptibility

**Fig. 2:**
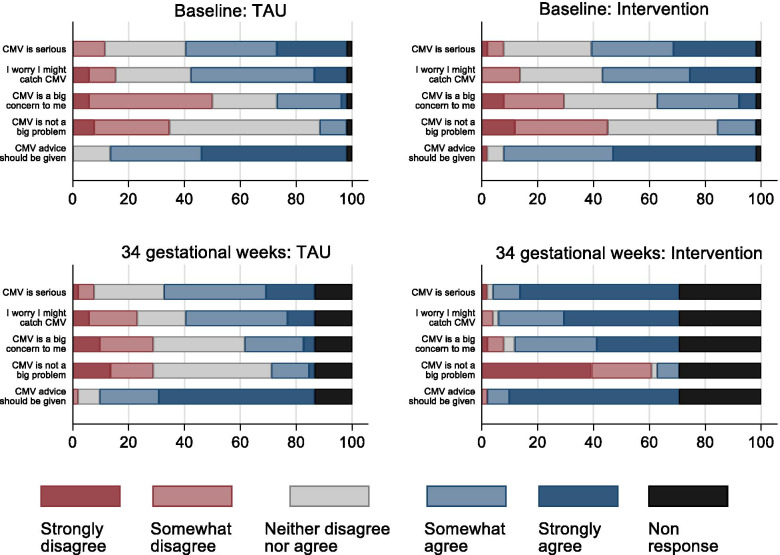
Attitudes of pregnant women toward the severity of CMV and their susceptibility to CMV at baseline and 34 gestational weeks in the treatment as usual group and the intervention group. CMV Cytomegalovirus; INT Intervention group; TAU Treatment As Usual group; Intention to treat analyses between intervention and treatment as usual groups at baseline (pre-intervention) and at 34 weeks gestation (post-intervention) and within intervention and treatment as usual groups comparisons between baseline and 34 weeks gestation

At baseline, participants’ perceptions about the severity of CMV and susceptibility to CMV were similar in the intervention and treatment as usual groups, Fig. 2. After the intervention (34 gestational weeks), a higher proportion of participants in the intervention group were likely to consider CMV to be serious and themselves personally susceptible to CMV, and to agree that advice about CMV should be given to pregnant women, compared to before the intervention (at baseline), Fig. 2. In contrast, the attitudes of pregnant women towards CMV in the treatment as usual group were similar at baseline and 34 gestational weeks, Fig. 2.

### Risk behaviours for CMV

At baseline, participants in the treatment as usual and intervention groups reported similar engagement with activities which might expose them to saliva or urine of children, for example commonly reporting eating left-over food from a child’s plate, Table 3. Within the intervention group, women reported eating left-over food, drinking from a child’s cup or kissing their child directly on the lips, less frequently after the intervention compared to before the intervention, Table 3. Differences in behaviours of women in the treatment as usual group were also observed between baseline and 34 gestational weeks, Table 3. Despite some differences in the frequency at which participants engaged in these activities in both groups, there was a difference between the two groups at 34 gestational weeks, with women in the intervention group reporting eating left-over food and kissing on the lips less frequently than women in the treatment as usual group, Table 3.

**Table 3:**
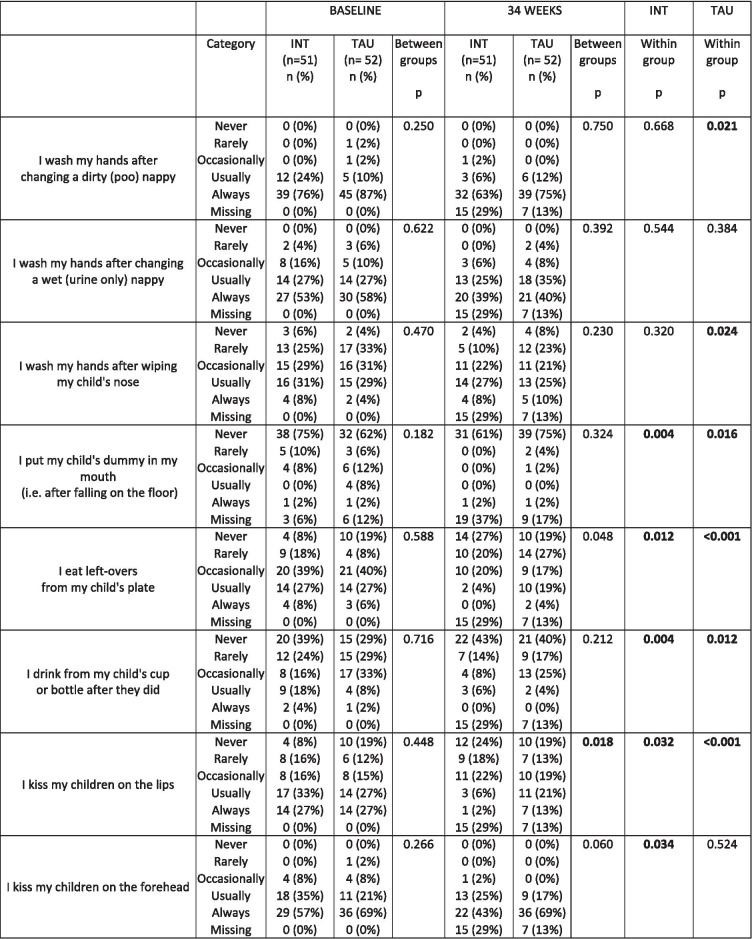
Engagement with activities that potentially expose women to saliva or urine of children

*INT* Intervention group; *TAU* Treatment As Usual group; Intention to treat analysis between intervention and treatment as usual groups at baseline (pre-intervention) and at 34 gestational weeks (post-intervention) and within intervention and treatment as usual groups at baseline and 34 gestational weeks

### Acceptability of educational intervention

Participants in the intervention group responded positively to the educational film, reporting that they felt motivated to change activities and felt confident that they could do so and would recommend the film to friends, Table 4.

**Table 4:**
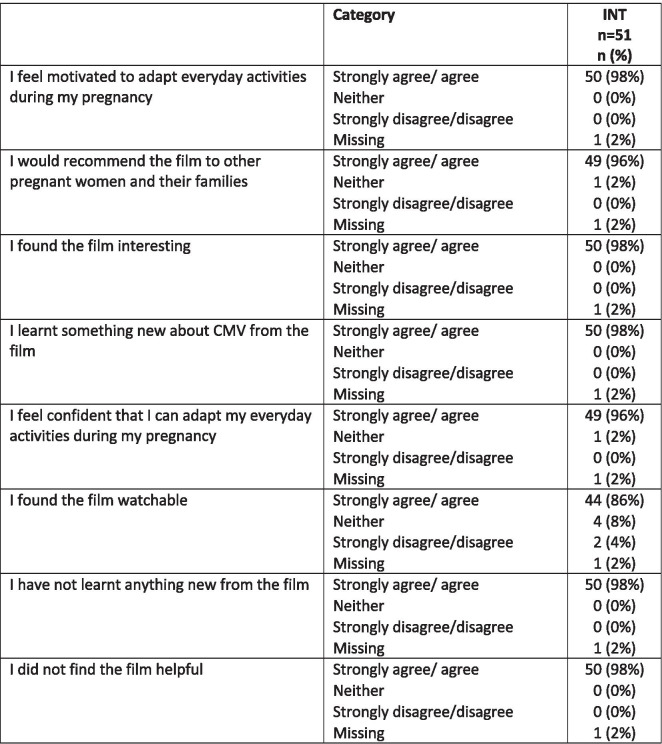
Reported responses to the educational intervention from participants in the intervention group

*INT* Intervention group; *CMV* Cytomegalovirus

### Anxiety, depression

There were no significant differences observed between scores on the Kessler Psychological Distress Scale or the Edinburgh Postnatal Depression Scale between the intervention and treatment as usual groups at baseline or at 34 weeks (Supplementary material 8; table 1).

### Seroconversion

Seroconversion between the end of the first trimester (baseline) and 34 gestational weeks was 4.55% in the intervention group and 4.65% in the treatment as usual group. There was one newborn infant, born to a mother in the intervention group who had seroconverted during pregnancy, who tested CMV PCR positive in urine at birth and therefore had congenital infection. The infant had no clinical features of cCMV and no treatment was required. The infant remained well with no clinical features of congenital CMV at 12 months of age.

## Discussion

This feasibility study demonstrates that recruitment to a future randomised controlled trial investigating the efficacy of a film-based educational intervention in reducing the risk of acquiring CMV infection in pregnancy would be feasible and has generated essential data upon which to design and power a larger RCT. This single-centre randomised controlled trial has shown that digital antenatal education about CMV is acceptable and accessible to pregnant women and does increase knowledge about CMV, change attitudes towards personal susceptibility and severity, and that pregnant women were willing to adopt risk-reducing behaviour change to reduce exposure to saliva and urine of young children. A future large multi-centre randomised controlled trial would be needed to determine whether such changes in knowledge, attitudes and behaviour would have an impact on seroconversion in pregnancy and therefore prevention of congenital CMV.

In this feasibility study, we have been able to identify factors which would be crucial to the design of a multi-centre randomised controlled trial. To determine the efficacy of an educational intervention, it is necessary to identify and enrol seronegative women in order to demonstrate seroconversion – thus acquisition of infection. We have shown that testing for CMV serology is highly acceptable to pregnant women in the first trimester of pregnancy; 2.86% (n = 144) of women declined testing for CMV antibodies, suggesting that the vast majority of women would be willing to be screened for CMV infection in pregnancy, in the NHS setting, and is consistent with that reported in other studies [20].

Multiparous seronegative women who have young children are at the highest risk of acquiring infection and transmitting this to their fetus, therefore these women would be the target population for future studies. We have demonstrated the challenges in identifying and enrolling this target population. A large number of women were ineligible for the study (n = 2320; 58.4%) because they were primiparous (this was their first pregnancy) and of those who were multiparous, a further 431 women were excluded because they did not have a child < 4 years of age. Together with the women who were ineligible for other reasons (n = 202) or for whom no sample was obtained (n = 13), only 878 (22%) of the 3975 women approached had a blood sample for CMV screening obtained. These factors are critical to take into account when designing and assessing the feasibility of future studies.

Of the women who consented for CMV screening, 43% were seronegative and therefore at risk of primary CMV infection and eligible for the study, and 57% of women were seropositive. The proportion of women who were seropositive varied considerably with ethnicity. The seropositivity in white women of 39% is similar to that seen in previous studies (45.9% Tookey, 1992; 49% Pembrey, 2013) [21, 22]. However, we found lower seropositivity in women from South Asian ethnicity (78%) compared to that seen in the cohort of pregnant women in Bradford (89%—98%) [22] and higher seropositivity in black women (94%) than has been observed in a population of women attending antenatal care in London in the 1990s (77%) [21]. Both Tookey et al. and Pembrey et al*.* found place of birth, as well as ethnicity to be important in seroprevalence, with British born women less likely to be seropositive [21, 22]. We did not collect information about place of birth and so were unable to investigate this aspect. Because of the eligibility requirements of the studies being recruited for, we only screened women living with a child aged less than four years, which may mean that this population is not completely representative of the pregnant population as a whole, but does represent women who are likely to be at the highest risk of infection in pregnancy.

A total of ten women (1.16%) had evidence indicating recent primary CMV infection within the first trimester of pregnancy, this is higher than that observed in an unselected population in a single centre in France (0.42% seroconversion) [20], but consistent with proportions seen in a population of women in Italy who had a young child or worked with young children (1.2%) [23]. Although this is a small proportion of women, this results in a large number of infants born each year with CMV. Vertical transmission in the first trimester of pregnancy is estimated at 36.8% with nearly 20% of fetuses from these women showing evidence of being affected by CMV [24]. Without interventions to reduce the risk of acquisition of CMV or transmission of CMV, these infants will continue to acquire CMV and a significant proportion of them continue to suffer long term adverse sequelae as a result of congenital CMV infection.

As well as generating essential data to inform a future larger study, we have also been able to describe important differences in knowledge about CMV, perceived severity, susceptibility and CMV risk reducing behaviour of pregnant women in the two study groups before the intervention in early pregnancy and at 34 gestational weeks. By collecting post-intervention data at 34 gestational weeks, we are able to show that these differences were evident even at the end pregnancy, suggesting that women were able to sustain these changes throughout pregnancy.

Before the intervention, most women were unfamiliar with CMV. Previous studies have also shown that only a minority of pregnant women have heard of CMV: 16% in an Australian study,[25] 18% in a Japanese study [26] and 20% in two separate studies in Singapore [27], and the US [28], and that the level of knowledge about CMV is less than for other conditions which affect newborn infants [25, 26, 28, 29]. Despite the fact that CMV is the most common congenital infection in the UK, pregnant women in our study were also less knowledgeable about CMV than other conditions affecting newborns. In our study, 34.7% of women reported being ‘somewhat’ or ‘very’ familiar, a higher proportion than in other studies. This may reflect volunteer bias in which those individuals who are better informed about CMV are more likely to take part in research about it, or it may have been a product of the screening process in which it was necessary to provide some information about CMV in the process of obtaining consent for serological screening.

Participants in the intervention group showed a greater awareness of the ways in which CMV can be transmitted and ways in which congenital CMV can affect children following the intervention, at 34 gestational weeks, compared to those women in the treatment as usual group. This is in agreement with the study by Price et al., who also included change in knowledge as an outcome following an antenatal educational intervention [12].

The ultimate aim of a CMV educational intervention in pregnancy is not acquisition of facts, but rather to modify behaviours that would place a woman at increased risk of exposure to CMV. In agreement with other studies [12, 20, 23, 30–32], we found that an educational intervention in pregnancy was associated with a reduction in the frequency of activities which could expose women to saliva and urine of young children, compared to before the intervention and compared to the treatment as usual group, specifically a reduction in participants eating leftovers from their child’s plate and kissing their child on the lips. These behaviours have previously been identified as being most difficult to change [33]. These changes in reported behaviours may relate to the change in the perception of severity and susceptibility which was seen in the intervention group; change in perception of severity of the condition and an individual’s susceptibility to it has been shown to be an important mediator of behaviour change [32].

As far as possible, we wanted to have a single intervention early in pregnancy in order to create circumstances as similar as possible to clinical practice, and we therefore provided no reminders to participants about risk reduction, we did not ask them about their behaviours between the first appointment and the questionnaire at 34 weeks and we did not use any objective measures of adherence which is in contrast to some other studies [30, 31, 34]. Whilst all of these measures were important to our ultimate goal of investigating an intervention which would have clinical utility in a routine setting, there are also limitations associated with this approach. Self-reported behaviour may not be the same as actual behaviour, especially when asking participants about their activities over a prolonged period. This may particularly be the case for those behaviours for which there is a perceived ‘right’ answer, for example washing hands after changing a nappy. We were unable to completely simulate real life conditions; in order to screen for the serostatus of potential participants it was necessary to provide some information about CMV which caused many of the participants to seek further information. This may have led to our whole study population being better informed about CMV than the general population and may have limited our ability to detect differences between the groups—although this would have led to an underestimation of the effect of the intervention and if such an intervention were used in routine care there might be an even greater impact on behaviours.

In this study we used a film as our educational intervention that had been designed in partnership with pregnant women and families of affected children. The feedback we received from study participants suggests that this was highly accessible and acceptable to them. Importantly, participants in the intervention group had similar scores on a global measure of distress and on a screening tool designed to identify individuals at risk of perinatal depression compared to those in the treatment as usual group – both pre- and post-intervention.

This study confirmed a finding which has been shown in repeated studies which is that pregnant women want to know about CMV and are often shocked that this has not been discussed with them before [26, 27, 34]. This reinforces the importance of a future large trial to determine the efficacy of an educational intervention to reduce the risk of CMV acquisition in pregnancy and the optimal implementation strategy for CMV antenatal education in routine clinical practice.

## Conclusions

We have demonstrated that a randomised controlled trial of a film-based educational intervention is feasible in the UK and generated essential data upon which to power such studies. This single-centre randomised controlled trial has also shown that the intervention was associated with differences in knowledge, attitudes and behaviours before and after the intervention. This gives confidence that it may be possible to reduce the risk of acquisition of CMV in pregnancy using a film-based educational intervention. The efficacy of this needs to be tested in a future multi-centre randomised controlled trial.

## Supplementary Information

**Additional file 1:** Description of digital educational intervention. Table outlining the timings and content of the digital educational intervention.

**Additional file 2:** Pre-intervention questionnaire (intervention and treatment as usual groups). Questionnaire completed by all participants at baseline.

**Additional file 3: **Post-intervention questionnaire (intervention group). Questionnaire completed by participants in the intervention group after viewing the digital educational intervention.

**Additional file 4:** Post-intervention questionnaire (treatment as usual group). Questionnaire completed by participants in the treatment as usual group after viewing the educational slides about influenza vaccination.

**Additional file 5:** 34-week questionnaire intervention group. Questionnaire completed by participants in the intervention group at 34 weeks of gestation.

**Additional file 6:** 34-week questionnaire TAU group. Questionnaire completed by participants in the treatment as usual group at 34 weeks of gestation.

**Additional file 7: Supplementary Figure S1**. Graphs showing familiarity of participants with conditions affecting newborn infants.

**Additional file 8: Supplementary Table S1**. Table of anxiety and depression scores for intervention and treatment as usual groups at baseline and 34 weeks.

**Additional file 9:** CONSORT extension for pilot and feasibility studies. This is the CONSORT extension checklist for pilot and feasibility studies. 

## Data Availability

The datasets analysed during the current study are available from the corresponding author on reasonable request.

## Abbreviations

CMV: Cytomegalovirus
cCMV: Congenital cytomegalovirus
ITT: Intention To Treat
PCR: Polymerase chain reaction
PP: Per protocol
RACE-FIT: Reducing Acquisition of CMV through antenatal Education study
RCT: Randomised controlled trial
TAU: Treatment as usual
UK: United Kingdom

## References

[1] Griffiths PD, Baboonian C, Rutter D, Peckham C (1991). Congenital and maternal cytomegalovirus infections in a London population. Br J Obstet Gynaecol.

[2] Dollard SC, Grosse SD, Ross DS (2007). New estimates of the prevalence of neurological and sensory sequelae and mortality associated with congenital cytomegalovirus infection. Rev Med Virol.

[3] Mussi-Pinhata MM, Yamamoto AY, Moura Brito RM, de Lima Isaac M, de Carvalho e Oliveira PF, Boppana S, et al. Birth prevalence and natural history of congenital cytomegalovirus infection in a highly seroimmune population. Clin Infect Dis. 2009;49(4):522–8. 10.1086/600882 PMC2778219 19583520

[4] Kenneson A, Cannon MJ (2007). Review and meta-analysis of the epidemiology of congenital cytomegalovirus (CMV) infection. Rev Med Virol.

[5] Staras SAS, Dollard SC, Radford KW, Flanders KW, Pass RF, Cannon MJ (2006). Seroprevalence of Cytomegalovirus Infection in the United States, 1988–1994. Clin Infect Dis.

[6] Marsico C, Kimberlin DW (2017). Congenital Cytomegalovirus infection: advances and challenges in diagnosis, prevention and treatment. Ital J Pediatr.

[7] Morton CC, Nance WE (2006). Newborn Hearing Screening- A Silent Revolution. N Engl J Med.

[8] Committee NS. Newborn screening for cytomegalovirus. 2017.

[9] Barber V, Calvert A, Vandrevala T, Star C, Khalil A, Griffiths P, Heath PT, Jones CE (2020). Prevention of Acquisition of Cytomegalovirus Infection in Pregnancy Through Hygiene-based Behavioral Interventions: A Systematic Review and Gap Analysis. Pediatr Infect Dis J.

[10] Vandrevala T, Barber V, Calvert A, Star C, Khalil A, Griffiths P, Heath PT, Jones CE. Understanding pregnant women’s readiness to engage in risk-reducing measures to prevent infections during pregnancy. J Health Psychol. 2019. 10.1177/1359105319884609 10.1177/1359105319884609 31686538

[11] Vandrevala T, Barber V, Mbire-Chigumba E, Calvert A, Star C, Khalil A, et al. Parenting a child with congenital cytomegalovirus infection: a qualitative study. BMJ Paediatr Open. 2020;4(1):e000844. 10.1136/bmjpo-2020-000844 PMC7662527 33225083

[12] Price SM, Bonilla E, Zador P, Levis DM, Kilgo CL, Cannon MJ. Educating women about congenital cytomegalovirus: assessment of health education materials through a web based survey. BMC Women’s Health. 2014;14(144). 10.1186/s12905-014-0144-3. 10.1186/s12905-014-0144-3 PMC4260245 25433837

[13] R. K. Kessler Psychological Distress Scale (K10) [Available from: https://www.tac.vic.gov.au/files-to-move/media/upload/k10_english.pdf.

[14] Cox JL, Holden JM, Sagovsky R. Edinburgh Postnatal Depression Scale. Available from: https://www.fresno.ucsf.edu/pediatrics/downloads/edinburghscale.pdf. 10.1192/bjp.150.6.782 3651732

[15] White IR, Horton NJ, Carpenter J, Pocock SJ. Strategy for intention to treat analysis in randomised trials with missing outcome data. Br Med J. 2011;342(d40). 10.1136/bmj.d40. 10.1136/bmj.d40 PMC3230114 21300711

[16] Hess S (2017). Randomization inference with Stata: A guide and software. Stand Genomic Sci.

[17] Kaiser J (2007). An exact and a Monte Carlo proposal to the Fisher-Pitman permutation tests for paired replicates and for independent samples. Stand Genomic Sci.

[18] Harris T (2013). Exact Wilcoxon signed-rank and Wilcoxon Mann-Whitney ranksum tests. Stand Genomic Sci.

[19] Chatfield M, Mander A (2009). The Skillings-Mack test (Friedman test when there are missing data). Stand Genomic Sci.

[20] Vauloup-Fellous C, Picone O, Cordier AG, Parent-du-Chatelet I, Senat MV, Frydman R (2009). Does hygiene counseling have an impact on the rate of CMV primary infection during pregnancy? Results of a 3-year prospective study in a French hospital. J Clin Virol.

[21] Tookey PAAA, Peckham C (1992). Cytomegalovirus prevalence in pregnant women: the influence of parity. Arch Dis Child.

[22] Pembrey L, Raynor P, Griffiths P, Chaytor S, Wright J, Hall AJ. Seroprevalence of cytomegalovirus, Epstein Barr virus and varicella zoster virus among pregnant women in Bradford: a cohort study. PLoS ONE. 2013; 8(11):e81881. 10.1371/journal.pone.0081881 PMC3842274 24312372

[23] Revello MG, Tibaldi C, Masuelli G, Frisina V, Sacchi A, Furione M (2015). Prevention of Primary Cytomegalovirus Infection in Pregnancy. EBioMedicine.

[24] Chatzakis C, Ville Y, Makrydimas G, Dinas K, Zavlanos A, Sotiriadis A. Timing of primary maternal cytomegalovirus infection and rates of vertical transmission and fetal consequences. Am J Obstet Gynecol. 2020; 223(6):870–83 e11. 10.1016/j.ajog.2020.05.038 32460972

[25] Lazzaro A, Vo ML, Zeltzer J, Rawlinson W, Nassar N, Daly K (2019). Knowledge of congenital cytomegalovirus (CMV) in pregnant women in Australia is low, and improved with education. Aust N Z J Obstet Gynaecol.

[26] Morioka I, Sonoyama A, Tairaku S, Ebina Y, Nagamata S, Morizane M (2014). Awareness of and knowledge about mother-to-child infections in Japanese pregnant women. Congenit Anom (Kyoto).

[27] Lim SL, Tan WC, Tan LK (2012). Awareness of and attitudes toward congenital cytomegalovirus infection among pregnant women in Singapore. Int J Gynaecol Obstet.

[28] Jeon J, Victor M, Adler SP, Arwady A, Demmler G, Fowler K (2006). Knowledge and awareness of congenital cytomegalovirus among women. Infect Dis Obstet Gynecol.

[29] Cannon MJ (2009). Congenital cytomegalovirus (CMV) epidemiology and awareness. J Clin Virol.

[30] Adler SP, Finney JW, Manganello AM, Best AM (1995). Prevention of chid-to-mother transmission of cytomegalovirus by changing behaviors: a randomized controlled trial. Pediatr Infect Dis J.

[31] Adler SP, Finney JW, Manganello AM, Best AM (2004). Prevention of child-to-mother transmission of cytomegalovirus among pregnant women. J Pediatr.

[32] Hughes BL, Gans KM, Raker C, Hipolito ER, Rouse DJ (2017). A Brief Prenatal Intervention of Behavioral Change to Reduce the Risk of Maternal Cytomegalovirus: A Randomized Controlled Trial. Obstet Gynecol.

[33] Thackeray R, Magnusson BM. Women’s attitudes toward practicing cytomegalovirus prevention behaviors. Prev Med Rep. 2016;4:517–24. 10.1016/j.pmedr.2016.09.008. 10.1016/j.pmedr.2016.09.008 PMC5061468 27747148

[34] Finney JW, Miller KM, Adler SP (1993). Changing Protective And Risky Behaviors To Prevent Child-To-Parent Transmission of Cytomegalovirus. J Appl Behav Anal.

