# Supplementary material for: Changing knowledge, attitudes and behaviours towards cytomegalovirus in pregnancy through film-based antenatal education: a feasibility randomised controlled trial of a digital educational intervention
Source: BMC Pregnancy Childbirth. 2021 Aug 18;21:565. doi: 10.1186/s12884-021-03979-z (PMC8375137; doi:10.1186/s12884-021-03979-z)
Supplement: Supplementary file 1 — Additional file 1: Description of digital educational intervention. Table outlining the timings and content of the digital educational intervention. [file 12884_2021_3979_MOESM1_ESM.docx]

|  | **Timing** | **Content** |
| --- | --- | --- |
| Section 1: Introduction | 0.00-1.06 | Introduction to pregnancy as a time where changes can make a difference to the future infant |
| Section 2: Basics of CMV | 1.07- 2.18 | Brief description of numbers infected and ways in which infants can be affected |
| Section 3: Impact of CMV on families | 2.19-5.39 | Stories from families with affected children about the impact it has had on their lives |
| Section 4: Transmission of CMV | 5.40-6.04 | Information about how CMV is transmitted and why people in contact with young children are at greater risk |
| Section 5: Risk reduction methods | 6.05-7.36 | Advice about how to reduce the risk of transmission |
| Section 6: Call to action | 7.37- 8.45 | Personal commentary from families about the importance of being empowered by knowledge in pregnancy |
| Section 7: Application | 8.46-9.30 | Comments from pregnant women about changes they will make in pregnancy to reduce the risk of infection |

**Summary of the digital educational intervention used in the RACE FIT study**
