# Supplementary material for: Changing knowledge, attitudes and behaviours towards cytomegalovirus in pregnancy through film-based antenatal education: a feasibility randomised controlled trial of a digital educational intervention
Source: BMC Pregnancy Childbirth. 2021 Aug 18;21:565. doi: 10.1186/s12884-021-03979-z (PMC8375137; doi:10.1186/s12884-021-03979-z)
Supplement: Supplementary file 2 — Additional file 2: Pre-intervention questionnaire (intervention and treatment as usual groups). Questionnaire completed by all participants at baseline. [file 12884_2021_3979_MOESM2_ESM.docx]

Single (never married)

Married/civil partner

Separated

Widowed

Divorced

Prefer not to say

RACEFIT

Questionnaire_PRE_INT

V2.0, 05/12/18

**Q2:** What is your marital status?

18-25 years

26-30 years

31-34 years

35-40 years

41-45 years

>45 years

**Q1:** How old are you?

**Firstly, we would like to ask some questions about you and your pregnancy:**

Thank you for agreeing to take part in this research study. Over the next 20-30 minutes you will be asked a series of questions. Please try to answer every question, if you need any help entering your answers on the ipad, please do ask one of the study team for help.

**Pre-intervention questionnaire (intervention and treatment as usual groups)**

**RACE FIT study**

**Q3:** How would you describe your ethnicity? Please tick the option that best applies to you.

RACEFIT

Questionnaire_PRE_INT

V2.0, 05/12/18

**Asian/Asian British:**

Indian

Pakistani

Bangladeshi

Any other Asian background, write in:

**Mixed/multiple ethnic groups:**

White and Black Caribbean

White & Black African

White and Asian

Any other Mixed/multiple ethnic background, write in:

**White:**

English/Welsh/Scottish/Northern Irish/British

Irish

Gypsy or Irish Traveller

Any other white background, write in:

RACEFIT

Questionnaire_PRE_INT

V2.0, 05/12/18

If you were not born in the UK, which country were you born in?

I was born in the UK

Less than 5 years

5-15 years

More than 15 years

**Q4:** How long have you lived in the UK? Please tick the best option.

**Other ethnic group:**

Arab

Any other ethnic group, write in:

**Black/African/Caribbean/Black British:**

African

Caribbean

Any other Black/African/Caribbean background, write in:

**Q7:** How many children do you have under the age of four years? (i.e. up to the age of their fourth birthday)

1 (this is my first pregnancy)

2

3

4

5

6

More than 6

1

2

3

More than 3

**Q6:** How many times have you been pregnant?

GCSE/BTEC or equivalent

A-levels, Scottish Highers or equivalent

Postgraduate Certificate, Diploma or equivalent

Bachelor of Science, Bachelor of Arts or equivalent

Masters degree or equivalent

PhD or equivalent

**Q5:** What is your highest qualification?

RACEFIT

Questionnaire_PRE_INT

V2.0, 05/12/18

RACEFIT

Questionnaire_PRE_INT

V2.0, 05/12/18

Yes No

**Q10:** If no, do you intend to have the flu vaccine?

Yes No

**Q9:** Have you already had the flu vaccine in this pregnancy?

No

Yes, as a childminder

Yes, in a nursery/pre-school setting

Yes, in a primary school

Yes, in a secondary school

Yes, in a neonatal unit

Yes, in a hospital (not a neonatal unit)

Yes, in afterschool activities

Yes, in another setting. Please specify:

**Q8:** Do you work regularly with children as part of your job?

|  | 1 | 2 | 3 | 4 | 5 |
| --- | --- | --- | --- | --- | --- |
| I don’t think I need this vaccine |  |  |  |  |  |
| I am concerned about the discomfort of the vaccine |  |  |  |  |  |
| My midwife, GP or obstetrician advised that I did not have this vaccine |  |  |  |  |  |
| My family/friends advised against having this vaccine |  |  |  |  |  |
| I am worried about information I have seen in the media about this vaccine |  |  |  |  |  |
| I don’t believe the vaccine is effective |  |  |  |  |  |
| I am concerned about potential side effects for my baby |  |  |  |  |  |
| I am concerned about potential side effects for me |  |  |  |  |  |
| I didn’t have enough information to decide whether or not to have this vaccine |  |  |  |  |  |
| Religious or other convictions |  |  |  |  |  |

RACEFIT

Questionnaire_PRE_INT

V2.0, 05/12/18

**Q11:** If no, why do you not intend to have the flu vaccine? Please indicate how much you agree with each of the following statements using a 1-5 scale where 1 is not at all important and 5 is very important.

If there were other reasons why you did not have the flu vaccine please state them in the box below:

**Q12:** Do you intend to have the whooping cough vaccine?

Yes No

|  | 1 | 2 | 3 | 4 | 5 |
| --- | --- | --- | --- | --- | --- |
| I don’t think I need this vaccine |  |  |  |  |  |
| I am concerned about the discomfort of the vaccine |  |  |  |  |  |
| My midwife, GP or obstetrician advised that I did not have this vaccine |  |  |  |  |  |
| My family/friends advised against having this vaccine |  |  |  |  |  |
| I am worried about information I have seen in the media about this vaccine |  |  |  |  |  |
| I don’t believe the vaccine is effective |  |  |  |  |  |
| I am concerned about potential side effects for my baby |  |  |  |  |  |
| I am concerned about potential side effects for me |  |  |  |  |  |
| I didn’t have enough information to decide whether or not to have this vaccine |  |  |  |  |  |
| Religious or other convictions |  |  |  |  |  |

RACEFIT

Questionnaire_PRE_INT

V2.0, 05/12/18

If there were other reasons why you did not have the whooping cough vaccine please state them in the box below:


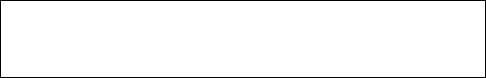


**Q13:** If no, why do you not intend to have the whooping cough vaccine? Please indicate how much you agree with each of the following statements using a 1-5 scale where 1 is not at all important and 5 is very important.

|  | *All of the time* | *Most of the time* | *Some of the time* | *A little of the time* | *None of the time* |
| --- | --- | --- | --- | --- | --- |
| In the past 4 weeks, about how often did you feel tired out for no good reason? |  |  |  |  |  |
| In the past 4 weeks, about how often did you feel nervous? |  |  |  |  |  |
| In the past 4 weeks, about how often did you feel so nervous that nothing could calm you down? |  |  |  |  |  |
| In the past 4 weeks, about how often did you feel hopeless? |  |  |  |  |  |
| In the past 4 weeks, about how often did you feel restless or fidgety?* |  |  |  |  |  |
| In the past 4 weeks, about how often did you feel so restless you could not sit still? |  |  |  |  |  |
| In the past 4 weeks, about how often did you feel depressed? |  |  |  |  |  |
| In the past 4 weeks, about how often did you feel that everything was an effort? |  |  |  |  |  |
| In the past 4 weeks, about how often did you feel so sad that nothing could cheer you up? |  |  |  |  |  |
| In the past 4 weeks, about how often did you feel worthless? |  |  |  |  |  |

*This point was excluded from analysis

As much as I always could

Not quite so much now

Definitely not so much now

Not at all

I have been able to laugh and see the funny side of things

**Q15:** Please tick the answer that comes closest to how you have felt in the past 7 DAYS, not just how you feel today.

In the past 7 days:

**Q14:** Over the past 4 weeks, how often have you experienced the following? Please select the response that most closely describes your experience for every question.

**Next we will ask you questions about how you have been feeling recently.**

RACEFIT

Questionnaire_PRE_INT

V2.0, 05/12/18

Yes, most of the time

Yes, sometimes

Not very often

No, not at all

Most of the time I have not been able to cope

Sometimes I haven’t been coping as well as usual

Most of the time I have coped

I have been coping as well as ever

Most of the time

Some of the time

Not very often

Never

As much as I ever did

Rather less than I used to

Definitely less than I used to

Hardly at all

Things have been getting on top of me

I have blamed myself unnecessarily when things went wrong

I have been so unhappy that I have had difficulty sleeping

I have looked forward with enjoyment to things

RACEFIT

Questionnaire_PRE_INT

V1.0, 5/6/18

|  | *All of the time* | *Most of the time* | *Some of the time* | *A little of the time* | *None of the time* |
| --- | --- | --- | --- | --- | --- |
| In the past 4 weeks, about how often did you feel tired out for no good reason? |  |  |  |  |  |
| In the past 4 weeks, about how often did you feel nervous? |  |  |  |  |  |
| In the past 4 weeks, about how often did you feel so nervous that nothing could calm you down? |  |  |  |  |  |
| In the past 4 weeks, about how often did you feel hopeless? |  |  |  |  |  |
| In the past 4 weeks, about how often did you feel restless or fidgety? |  |  |  |  |  |
| In the past 4 weeks, about how often did you feel so restless you could not sit still? |  |  |  |  |  |
| In the past 4 weeks, about how often did you feel depressed? |  |  |  |  |  |
| In the past 4 weeks, about how often did you feel that everything was an effort? |  |  |  |  |  |
| In the past 4 weeks, about how often did you feel so sad that nothing could cheer you up? |  |  |  |  |  |
| In the past 4 weeks, about how often did you feel worthless? |  |  |  |  |  |

I have been scared or panicky for no very good reason

|  | *Not at all true* | *Hardly true* | *Moderately true* | *Exactly true* |
| --- | --- | --- | --- | --- |
| I can always manage to solve difficult problems if I try hard enough |  |  |  |  |
| If someone opposes me, I can find the means and ways to get what I want |  |  |  |  |
| It is easy for me to stick to my aims and accomplish my goals |  |  |  |  |
| I am confident that I could deal efficiently with unexpected events |  |  |  |  |
| Thanks to my resourcefulness, I know how to handle unforeseen situations |  |  |  |  |
| I can solve most problems if I invest the necessary effort |  |  |  |  |
| I can remain calm when facing difficulties because I can rely on my coping abilities |  |  |  |  |
| When I am confronted with a problem, I can usually find several solutions |  |  |  |  |
| If I am in trouble, I can usually think of a solution |  |  |  |  |
| I can usually handle whatever comes my way |  |  |  |  |

I have felt sad or miserable

Very often

Sometimes

Hardly ever

Not at all

I have been anxious or worried for no good reason

Most of the time

Quite often

Only occasionally

Never

I have been scared or panicky for no very good reason

Quite a lot

Sometimes

Not much

Not at all

I have been so unhappy that I have been crying

Most of the time

Quite often

Only occasionally

Never

|  | *Not at all true* | *Hardly true* | *Moderately true* | *Exactly true* |
| --- | --- | --- | --- | --- |
| I can always manage to solve difficult problems if I try hard enough |  |  |  |  |
| If someone opposes me, I can find the means and ways to get what I want |  |  |  |  |
| It is easy for me to stick to my aims and accomplish my goals |  |  |  |  |
| I am confident that I could deal efficiently with unexpected events |  |  |  |  |
| Thanks to my resourcefulness, I know how to handle unforeseen situations |  |  |  |  |
| I can solve most problems if I invest the necessary effort |  |  |  |  |
| I can remain calm when facing difficulties because I can rely on my coping abilities |  |  |  |  |
| When I am confronted with a problem, I can usually find several solutions |  |  |  |  |
| If I am in trouble, I can usually think of a solution |  |  |  |  |
| I can usually handle whatever comes my way |  |  |  |  |

RACEFIT

Questionnaire_PRE_INT

V2.0, 05/12/18

**Q16**: Please tick the response that most closely describes your experience for every statement.

If you are experiencing low mood or the thought of harming yourself has occurred to you, please speak to your midwife, GP or obstetrician. If you would like to speak to someone today, please let a member of the research team know.

Quite often

Sometimes

Hardly ever

Never

The thought of harming myself has occurred to me

**Q.17:** Since you were approached about the RACE FIT study have you looked for information about CMV?

**Thank you for answering those questions. We will now ask some questions about infections in pregnancy.**

RACEFIT

Questionnaire_PRE_INT

V2.0, 05/12/18

Yes

No

**Q.18**: If yes, what sources of information did you use?

Books

Google search

CMV action

NHS Choices

Midwife/GP/Obstetrician

Other (please specify)

**Q19:** How familiar are you with the following conditions and infections? Please tick the response that corresponds to you the most.

|  | Not at all familiar | Somewhat familiar | Very familiar |
| --- | --- | --- | --- |
| Down syndrome |  |  |  |
| Cytomegalovirus |  |  |  |
| Toxoplasmosis |  |  |  |
| Listeria |  |  |  |
| Rubella |  |  |  |

**Q20:** How common do you think the following health conditions are in newborn babies?

|  | Very common | Fairly common | Not very common | Not common at all | Don’t know |
| --- | --- | --- | --- | --- | --- |
| Down syndrome |  |  |  |  |  |
| Cytomegalovirus |  |  |  |  |  |
| Toxoplasmosis |  |  |  |  |  |
| Listeria |  |  |  |  |  |
| Rubella |  |  |  |  |  |

RACEFIT

Questionnaire_PRE_INT

V2.0, 05/12/18

**Q21:** Rate how much you agree with each of the statements that relate to a particular infection – cytomegalovirus (CMV). Please tick the response that corresponds to you the most.

RACEFIT

Questionnaire_PRE_INT

V2.0, 05/12/18

|  | *Strongly agree* | *Somewhat agree* | *Neither agree or disagree* | *Somewhat disagree* | *Strongly disagree* |
| --- | --- | --- | --- | --- | --- |
| CMV is preventable |  |  |  |  |  |
| CMV can be spread through saliva |  |  |  |  |  |
| CMV can be spread through urine |  |  |  |  |  |
| CMV can be spread through faeces (poo) |  |  |  |  |  |
| CMV can be spread through hugging or cuddling |  |  |  |  |  |
| CMV can spread by casual contact with someone |  |  |  |  |  |
| Down Syndrome affects babies as often as CMV infection |  |  |  |  |  |
| CMV can cause hearing loss in a new born baby |  |  |  |  |  |
| CMV can cause intellectual disability in a newborn baby |  |  |  |  |  |
| CMV can cause physical disability in a newborn baby |  |  |  |  |  |
| CMV can cause heart defects in a newborn baby |  |  |  |  |  |
| CMV can cause hearing loss in a pregnant woman |  |  |  |  |  |
| CMV is serious |  |  |  |  |  |
| I worry that I might catch CMV and it would affect my baby |  |  |  |  |  |
| CMV is a big concern to me |  |  |  |  |  |
| CMV is not a big problem |  |  |  |  |  |
| Pregnant women should be given advice about preventing CMV infection during pregnancy |  |  |  |  |  |

**Q22:** How often would you do each of the following activities? Please select the best response for you.

|  | *Always* | *Usually* | *Occasionally* | *Rarely* | *Never* |
| --- | --- | --- | --- | --- | --- |
| I wash my hands after changing a dirty (poo) nappy |  |  |  |  |  |
| I wash my hands after changing a wet nappy (urine only) |  |  |  |  |  |
| I wash my hands after wiping my child’s nose |  |  |  |  |  |
| I put my child’s dummy in my mouth (for example, if fallen on floor) |  |  |  |  |  |
| I eat left-overs from my child’s plate |  |  |  |  |  |
| I drink from my child’s cup or bottle after they have had a drink from it |  |  |  |  |  |
| I kiss my children on the lips |  |  |  |  |  |
| I kiss children on the forehead |  |  |  |  |  |

**Thank you, you have reached the end of the questions!**

**Finally, we would like you to think about day to day activities that we all do.**
