# Supplementary material for: Changing knowledge, attitudes and behaviours towards cytomegalovirus in pregnancy through film-based antenatal education: a feasibility randomised controlled trial of a digital educational intervention
Source: BMC Pregnancy Childbirth. 2021 Aug 18;21:565. doi: 10.1186/s12884-021-03979-z (PMC8375137; doi:10.1186/s12884-021-03979-z)
Supplement: Supplementary file 3 — Additional file 3: Post-intervention questionnaire (intervention group). Questionnaire completed by participants in the intervention group after viewing the digital educational intervention. [file 12884_2021_3979_MOESM3_ESM.docx]

Post-intervention questionnaire – intervention group only, immediately after intervention

Thank you for watching the film, we would now like to ask you a few questions about what you thought about the film. This questionnaire will take 5-7 min to complete.

**Q1** Please let us know what you thought about the film, select the best answer:

|  | Strongly Disagree | Disagree | Neither | Agree | Strongly Agree |
| --- | --- | --- | --- | --- | --- |
| I found the film watchable |  |  |  |  |  |
| I found the film interesting |  |  |  |  |  |
| I feel motivated to adapt everyday activities during my pregnancy |  |  |  |  |  |
| I feel confident that I can adapt my everyday activities during my pregnancy |  |  |  |  |  |
| I learnt something new about CMV from the film |  |  |  |  |  |
| I did not find the film helpful |  |  |  |  |  |
| I have not learnt anything new from the film |  |  |  |  |  |
| I would recommend the film to other pregnant women and their families |  |  |  |  |  |

**Q2** List the most positive aspect(s):

1.

2.

3.

**Q3** List the most negative aspect(s):

1.

2.

3.

**Q4** List the most useful parts of the film:

1.

2.

3.

**Q5** What would you change in the film?

1.

2.

3.

**Q6** Please select the best answer that applies to you:

|  | *Strongly agree* | *Somewhat agree* | *Disagree* | *Strongly disagree* | *Neither agree or disagree* |
| --- | --- | --- | --- | --- | --- |
| I am motivated to do anything to keep my unborn baby safe |  |  |  |  |  |
| 9 months is a short time to make these changes |  |  |  |  |  |
| I can’t stop kissing my child on the lips for 9 months |  |  |  |  |  |
| Not eating the left-overs from my children’s plate is going to be difficult |  |  |  |  |  |
| I believe that kissing children is a way of showing affection |  |  |  |  |  |
| I am worried that changing my behaviours will have a negative impact on my child |  |  |  |  |  |
| It is too late in the pregnancy to change my behaviour |  |  |  |  |  |
| I fear that changing my behaviour will cause offence to others |  |  |  |  |  |
| I now feel confident to discuss CMV with other pregnant women |  |  |  |  |  |
| I now feel confident to discuss CMV with my partner |  |  |  |  |  |
| I now feel confident to discuss CMV with my family |  |  |  |  |  |

**Q7** How confident do you feel about being able to follow the behaviours listed below? Please tick the response that corresponds to you the most.

|  | *Always* | *Usually* | *Occasionally* | *Rarely* | *Never* |
| --- | --- | --- | --- | --- | --- |
| Avoiding kissing young children on the lips |  |  |  |  |  |
| Avoid eating or drinking anything that has been in my child’s mouth |  |  |  |  |  |
| Not putting a child’s dummy in your mouth |  |  |  |  |  |
| Washing your hands every time you come in contact with a child’s urine or saliva |  |  |  |  |  |

**Q8** I intend to follow the behaviours listed below during the duration of my pregnancy. Please tick the response that corresponds to you the most.

|  | *Strongly agree* | *Agree* | *Neither agree or disagree* | *Disagree* | *Strongly disagree* |
| --- | --- | --- | --- | --- | --- |
| Avoiding kissing young children on the lips |  |  |  |  |  |
| Avoid eating or drinking anything that has been in my child’s mouth |  |  |  |  |  |
| Not putting a child’s dummy in my mouth |  |  |  |  |  |
| Washing my hands every time I come in contact with a child’s urine or saliva |  |  |  |  |  |

Thank you, you have reached the end of the questions.
