# Supplementary material for: Changing knowledge, attitudes and behaviours towards cytomegalovirus in pregnancy through film-based antenatal education: a feasibility randomised controlled trial of a digital educational intervention
Source: BMC Pregnancy Childbirth. 2021 Aug 18;21:565. doi: 10.1186/s12884-021-03979-z (PMC8375137; doi:10.1186/s12884-021-03979-z)
Supplement: Supplementary file 4 — Additional file 4: Post-intervention questionnaire (treatment as usual group). Questionnaire completed by participants in the treatment as usual group after viewing the educational slides about influenza vaccination. [file 12884_2021_3979_MOESM4_ESM.pdf]

Post-intervention questionnaire – treatment as usual group only, immediately after intervention

Thank you for looking at the information about vaccines in pregnancy, we would now like to ask you a few questions about what you thought about the slide set.

Q1. Please select the best answer:

	Strongly Disagree	*Disagree*	*Neither*	*Agree*	*Strongly Agree*
I found the information easy to understand					
I found the information interesting					
I feel motivated to have the flu vaccine					
I intend to have the flu vaccine					
I intend to have the whooping cough vaccine					
I learnt something new about flu vaccination					
I would recommend the flu vaccine to other pregnant women					
The information provided to me influenced my decision to get vaccinated					
