# Supplementary material for: Changing knowledge, attitudes and behaviours towards cytomegalovirus in pregnancy through film-based antenatal education: a feasibility randomised controlled trial of a digital educational intervention
Source: BMC Pregnancy Childbirth. 2021 Aug 18;21:565. doi: 10.1186/s12884-021-03979-z (PMC8375137; doi:10.1186/s12884-021-03979-z)
Supplement: Supplementary file 5 — Additional file 5: 34-week questionnaire intervention group. Questionnaire completed by participants in the intervention group at 34 weeks of gestation. [file 12884_2021_3979_MOESM5_ESM.docx]

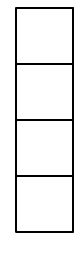


|  | 1 | 2 | 3 | 4 | 5 |
| --- | --- | --- | --- | --- | --- |
| I don’t think I need this vaccine |  |  |  |  |  |
| I am concerned about the discomfort of the vaccine |  |  |  |  |  |
| My midwife, GP or obstetrician advised that I did not have the vaccine |  |  |  |  |  |
| My family/friends advised against having this vaccine |  |  |  |  |  |
| I am worried about information I have seen in the media about this vaccine |  |  |  |  |  |
| I don’t believe the vaccine is effective |  |  |  |  |  |
| I am concerned about potential side effects for my baby |  |  |  |  |  |
| I am concerned about potential side effects for me |  |  |  |  |  |
| I didn’t have enough information to decide whether or not to have this vaccine |  |  |  |  |  |
| Religious or other convictions |  |  |  |  |  |

Yes No

**Q3**: If no, why did you not have the flu vaccine?

Please indicate how much you agree with each of the following statements using a 1-5 scale where 1 is not at all important and 5 is very important.

**34 week questionnaire (intervention group)**

**RACE FIT study**

Thank you for taking part in the RACE FIT study. We would like to ask you some questions about how you have felt during your pregnancy and explore how your daily activities might have changed during this time. The questionnaire will take approximately 10 minutes of your time. Please try to answer every question.

**Q2**: Did you receive the flu vaccine in this pregnancy?

Y

Y

M

M

D

D

**Q1**: What’s your estimated Date of Delivery (EDD) in your current pregnancy?

RACEFIT

Questionnaire_34 weeks_INT

V 2.0, 05.12.18

|  | 1 | 2 | 3 | 4 | 5 |
| --- | --- | --- | --- | --- | --- |
| I don’t think I need the vaccine |  |  |  |  |  |
| I am concerned about the discomfort of the vaccine |  |  |  |  |  |
| My midwife, GP or obstetrician advised that I did not have this vaccine |  |  |  |  |  |
| My family/friends advised against having this vaccine |  |  |  |  |  |
| I am worried about information I have seen in the media about this vaccine |  |  |  |  |  |
| I don’t believe the vaccine is effective |  |  |  |  |  |
| I am concerned about potential side effects for my baby |  |  |  |  |  |
| I am concerned about potential side effects for me |  |  |  |  |  |
| I didn’t have enough information to decide whether or not to have this vaccine |  |  |  |  |  |
| Religious or other convictions |  |  |  |  |  |

If there were other reasons why you did not have the whooping cough vaccine please state them in the box below:

If no, why did you not have the whooping cough vaccine?

Please indicate how much you agree with each of the following statements using a 1-5 scale where 1 is not at all important and 5 is very important.

Yes No

**Q4**: Did you receive the whooping cough vaccine in this pregnancy?

If there were other reasons why you did not have the flu vaccine please state them in the box below:

RACEFIT

Questionnaire_34 weeks_INT

V 2.0, 05.12.18

**Q6**: Please tick the answer that comes closest to how you have felt in the past 7 DAYS, not just how you feel today.

In the past 7 days:

I have been able to laugh and see the funny side of things

As much as I always could

Not quite so much now

Definitely not so much now

Not at all

|  | *All of the time* | *Most of the time* | *Some of the time* | *A little of the time* | *None of the time* |
| --- | --- | --- | --- | --- | --- |
| In the past 4 weeks, about how often did you feel tired out for no good reason? |  |  |  |  |  |
| In the past 4 weeks, about how often did you feel nervous? |  |  |  |  |  |
| In the past 4 weeks, about how often did you feel so nervous that nothing could calm you down? |  |  |  |  |  |
| In the past 4 weeks, about how often did you feel hopeless? |  |  |  |  |  |
| In the past 4 weeks, about how often did you feel restless or fidgety?* |  |  |  |  |  |
| In the past 4 weeks, about how often did you feel so restless you could not sit still? |  |  |  |  |  |
| In the past 4 weeks, about how often did you feel depressed? |  |  |  |  |  |
| In the past 4 weeks, about how often did you feel that everything was an effort? |  |  |  |  |  |
| In the past 4 weeks, about how often did you feel so sad that nothing could cheer you up? |  |  |  |  |  |
| In the past 4 weeks, about how often did you feel worthless? |  |  |  |  |  |

*This point was excluded from analysis

**Q5**: Over the past 4 weeks, how often have you experienced the following?

Please select the response that most closely describes your experience for every question.

RACEFIT

Questionnaire_34 weeks_INT

V 2.0, 05.12.18

As much as I ever did

Rather less than I used to

Definitely less than I used to

Hardly at all

I have blamed myself unnecessarily when things went wrong

I have looked forward with enjoyment to things

Quite a lot

Sometimes

Not much

Not at all

I have been scared or panicky for no very good reason

Very often

Sometimes

Hardly ever

Not at all

I have been anxious or worried for no good reason

RACEFIT

Questionnaire_34 weeks_INT

V 1.0, 05.06.18

Most of the time

Some of the time

Not very often

Never


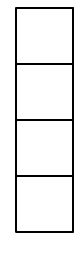


Most of the time I have not been able to cope

Sometimes I haven’t been coping as well as usual

Most of the time I have coped

I have been coping as well as ever

Most of the time

Sometimes

Not very often

Not at all

Most of the time

Quite often

Only occasionally

Never

Most of the time

Quite often

Only occasionally

Never

I have been so unhappy that I have been crying

I have felt sad or miserable

I have been so unhappy that I have had difficulty sleeping

Things have been getting on top of me

RACEFIT

Questionnaire_34 weeks_INT

V 2.0, 05.12.18

Quite often

Sometimes

Hardly ever

Never

|  | *Not at all true* | *Hardly true* | *Moderately true* | *Exactly true* |
| --- | --- | --- | --- | --- |
| I can always manage to solve difficult problems if I try hard enough |  |  |  |  |
| If someone opposes me, I can find the means and ways to get what I want |  |  |  |  |
| It is easy for me to stick to my aims and accomplish my goals |  |  |  |  |
| I am confident that I could deal efficiently with unexpected events |  |  |  |  |
| Thanks to my resourcefulness, I know how to handle unforeseen situations |  |  |  |  |
| I can solve most problems if I invest the necessary effort |  |  |  |  |
| I can remain calm when facing difficulties because I can rely on my coping abilities |  |  |  |  |
| When I am confronted with a problem, I can usually find several solutions |  |  |  |  |
| If I am in trouble, I can usually think of a solution |  |  |  |  |
| I can usually handle whatever comes my way |  |  |  |  |

**Q7**: Please tick the response that most closely describes your experience for every statement.

If you are experiencing low mood or the thought of harming yourself has occurred to you, please speak to your midwife, GP or obstetrician. If you would like to speak to someone today, please let a member of the research team know.

The thought of harming myself has occurred to me

RACEFIT

Questionnaire_34 weeks_INT

V 2.0, 05.12.18

|  | *Strongly agree* | *Somewhat agree* | *Neither agree or disagree* | *Somewhat disagree* | *Strongly disagree* |
| --- | --- | --- | --- | --- | --- |
| CMV is preventable |  |  |  |  |  |
| CMV can be spread through saliva |  |  |  |  |  |
| CMV can be spread through urine |  |  |  |  |  |
| CMV can be spread through faeces (poo) |  |  |  |  |  |
| CMV can be spread through hugging or cuddling |  |  |  |  |  |
| CMV can spread by casual contact with someone |  |  |  |  |  |
| Down Syndrome affects babies as often as CMV infection |  |  |  |  |  |
| CMV can cause hearing loss in a newborn baby |  |  |  |  |  |
| CMV can cause intellectual disability in a newborn baby |  |  |  |  |  |
| CMV can cause physical disability in a newborn baby |  |  |  |  |  |
| CMV can cause heart defects in a newborn baby |  |  |  |  |  |
| CMV can cause hearing loss in a pregnant woman |  |  |  |  |  |
| CMV is serious |  |  |  |  |  |
| I worry that I might catch CMV and it would affect my baby |  |  |  |  |  |
| CMV is a big concern to me |  |  |  |  |  |
| CMV is not a big problem |  |  |  |  |  |
| Pregnant women should be given advice about preventing CMV infection during pregnancy |  |  |  |  |  |

**Q8**: Regarding cytomegalovirus (CMV). Rate how much you agree with each of the statements. Please tick the response that corresponds to you the most.

RACEFIT

Questionnaire_34 weeks_INT

V 2.0, 05.12.18

|  | *Very easy* | *Easy* | *Neither easy nor difficult* | *Difficult* | *Impossible* |
| --- | --- | --- | --- | --- | --- |
| Washing my hands after changing a nappy |  |  |  |  |  |
| Washing my hands after wiping my child’s nose |  |  |  |  |  |
| Not putting my child’s dummy in my mouth (for example, if fallen on the floor) |  |  |  |  |  |
| Not eating left-overs from my child’s plate |  |  |  |  |  |
| Not drinking from my child’s cup or bottle after they have had a drink from it |  |  |  |  |  |
| Not kissing my child on the lips |  |  |  |  |  |

**Q10**: Please rate how difficult it has been for you to do each of the following activities to reduce the risk of catching CMV

|  | *Always* | *Usually* | *Occasionally* | *Rarely* | *Never* |
| --- | --- | --- | --- | --- | --- |
| Wash my hands after changing a dirty (poo) nappy |  |  |  |  |  |
| Wash hands after changing a wet nappy (urine only) |  |  |  |  |  |
| Wash hands after wiping my child’s nose |  |  |  |  |  |
| Put my child’s dummy in my mouth (for example, if fallen on floor) |  |  |  |  |  |
| Eat left-overs on my child’s plate |  |  |  |  |  |
| Drink from my child’s cup or bottle after they have had a drink from it |  |  |  |  |  |
| Kiss my children on the lips |  |  |  |  |  |
| Kiss children on the forehead |  |  |  |  |  |

**Q9**: Thinking back over the last 4 weeks of pregnancy, how often have you done each of the following activities?

Please tick the response that corresponds to you the most.

RACEFIT

Questionnaire_34 weeks_INT

V 2.0, 05.12.18

**Q11:** Have these changes become normal in your house now?

**Q13:** What has made it difficult to make these changes?

Yes No

Yes No

**Q12:** Has your partner also made these changes?

**Q14:** What has helped you make these changes?

**Q15:** What advice would you give to a close friend to help them make these changes?

RACEFIT

Questionnaire_34 weeks_INT

V 2.0, 05.12.18

**Q16**: Did you watch the RACE FIT film again?

Yes No

**Q17**: Did you share the film with anyone else?

Yes No

If yes, who did you show the film to?

Partner

Other family members

Friends who were pregnant

Other pregnant women taking part in the study

**Q18**: Have you looked for information about CMV from any sources?

Yes No

If yes, where have you looked for information about CMV?

Books

Google search

CMV action

NHS Choices

Asked Midwife/GP/Obstetrician

Other (please specify)

**Thank you, you have reached the end of the questions!**

RACEFIT

Questionnaire_34 weeks_INT

V 2.0, 05.12.18

**Q11:** Have these changes become normal in your house now?
