# Supplementary material for: Changing knowledge, attitudes and behaviours towards cytomegalovirus in pregnancy through film-based antenatal education: a feasibility randomised controlled trial of a digital educational intervention
Source: BMC Pregnancy Childbirth. 2021 Aug 18;21:565. doi: 10.1186/s12884-021-03979-z (PMC8375137; doi:10.1186/s12884-021-03979-z)
Supplement: Supplementary file 6 — Additional file 6: 34-week questionnaire TAU group. Questionnaire completed by participants in the treatment as usual group at 34 weeks of gestation. [file 12884_2021_3979_MOESM6_ESM.pdf]

34 week questionnaire (Treatment as usual groups)

RACE FIT study

Thank you for taking part in the RACE FIT study. We would like to ask you some questions about how you have felt during your pregnancy and explore how your daily activities might have changed during this time. The questionnaire will take approximately 10 minutes of your time. Please try to answer every question.

Q1: What's your estimated Date of Delivery (EDD) in your current pregnancy?

D D M M Y Y

Q2: Did you receive the flu vaccine in this pregnancy?

Yes ☐ No ☐

If no, why did you not have the flu vaccine?

Please indicate how much you agree with each of the following statements using a 1-5 scale where 1 is not at all important and 5 is very important.

	1	2	3	4	5
I don't think I need this vaccine					
I am concerned about the discomfort of the vaccine					
My midwife, GP or obstetrician advised that I did not have this vaccine					
My family/friends advised against having this vaccine					
I am worried about information I have seen in the media about this vaccine					
I don't believe the vaccine is effective					
I am concerned about potential side effects for my baby					
I am concerned about potential side effects for me					
I didn't have enough information to decide whether or not to have this vaccine					
Religious or other convictions					

If there were other reasons why you did not have the flu vaccine please state them in the box below:

Q3: Did you receive the whooping cough vaccine in this pregnancy?

Yes ☐ No ☐

If no, why did you not have the whooping cough vaccine?

Please indicate how much you agree with each of the following statements using a 1-5 scale where 1 is not at all important and 5 is very important.

	1	2	3	4	5
I don't think I need this vaccine					
I am concerned about the discomfort of the vaccine					
My midwife, GP or obstetrician advised that I did not have this vaccine					
My family/friends advised against having this vaccine					
I am worried about information I have seen in the media about this vaccine					
I don't believe the vaccine is effective					
I am concerned about potential side effects for my baby					
I am concerned about potential side effects for me					
I didn't have enough information to decide whether or not to have this vaccine					
Religious or other convictions					

If there were other reasons why you did not have the whooping cough vaccine please state them in the box below:

Q4: Over the past 4 weeks, how often have you experienced the following?

Please select the response that most closely describes your experience for every question.

	All of the time	*Most of the time*	*Some of the time*	*A little of the time*	*None of the time*
In the past 4 weeks, about how often did you feel tired out for no good reason?					
In the past 4 weeks, about how often did you feel nervous?					
In the past 4 weeks, about how often did you feel so nervous that nothing could calm you down?					
In the past 4 weeks, about how often did you feel hopeless?					
In the past 4 weeks, about how often did you feel restless or fidgety?*					
In the past 4 weeks, about how often did you feel so restless you could not sit still?					
In the past 4 weeks, about how often did you feel depressed?					
In the past 4 weeks, about how often did you feel that everything was an effort?					
In the past 4 weeks, about how often did you feel so sad that nothing could cheer you up?					
In the past 4 weeks, about how often did you feel worthless?					

*This point was excluded from analysis

Q5: Please tick the answer that comes closest to how you have felt in the past 7 DAYS, not just how you feel today.

In the past 7 days:

I have been able to laugh and see the funny side of things

As much as I always could	☐
Not quite so much now	☐
Definitely not so much now	☐
Not at all	☐

I have looked forward with enjoyment to things

- As much as I ever did ☐
- Rather less than I used to ☐
- Definitely less than I used to ☐
- Hardly at all ☐

I have blamed myself unnecessarily when things went wrong

- Most of the time ☐
- Some of the time ☐
- Not very often ☐
- Never ☐

I have been anxious or worried for no good reason

- Very often ☐
- Sometimes ☐
- Hardly ever ☐
- Not at all ☐

I have been scared or panicky for no very good reason

- Quite a lot ☐
- Sometimes ☐
- Not much ☐
- Not at all ☐

Things have been getting on top of me

- Most of the time I have not been able to cope ☐
- Sometimes I haven't been coping as well as usual ☐
- Most of the time I have coped ☐
- I have been coping as well as ever ☐

I have been so unhappy that I have had difficulty sleeping

- Most of the time ☐
- Sometimes ☐
- Not very often ☐
- Not at all ☐

I have felt sad or miserable

- Most of the time ☐
- Quite often ☐
- Only occasionally ☐
- Never ☐

I have been so unhappy that I have been crying

- Most of the time ☐
- Quite often ☐
- Only occasionally ☐
- Never ☐

The thought of harming myself has occurred to me

Quite often	☐
Sometimes	☐
Hardly ever	☐
Never	☐

If you are experiencing low mood or the thought of harming yourself has occurred to you, please speak to your midwife, GP or obstetrician. If you would like to speak to someone today, please let a member of the research team know.

Q6: Please tick the response that most closely describes your experience for every statement.

	Not at all true	*Hardly true*	*Moderately true*	*Exactly true*
I can always manage to solve difficult problems if I try hard enough				
If someone opposes me, I can find the means and ways to get what I want				
It is easy for me to stick to my aims and accomplish my goals				
I am confident that I could deal efficiently with unexpected events				
Thanks to my resourcefulness, I know how to handle unforeseen situations				
I can solve most problems if I invest the necessary effort				
I can remain calm when facing difficulties because I can rely on my coping abilities				
When I am confronted with a problem, I can usually find several solutions				
If I am in trouble, I can usually think of a solution				
I can usually handle whatever comes my way				

Q7: Regarding cytomegalovirus (CMV) Rate how much you agree with each of the statements. Please tick the response that corresponds to you the most.

	Strongly agree	*Somewhat agree*	*Neither agree or disagree*	*Somewhat disagree*	*Strongly disagree*
CMV is preventable					
CMV can be spread through saliva					
CMV can be spread through urine					
CMV can be spread through faeces (poo)					
CMV can be spread through hugging or cuddling					
CMV can spread by casual contact with someone					
Down Syndrome affects babies as often as CMV infection					
CMV can cause hearing loss in a newborn baby					
CMV can cause intellectual disability in a newborn baby					
CMV can cause physical disability in a newborn baby					
CMV can cause heart defects in a newborn baby					
CMV can cause hearing loss in a pregnant woman					
CMV is serious					
I worry that I might catch CMV and it would affect my baby					
CMV is a big concern to me					
CMV is not a big problem					
Pregnant women should be given advice about preventing CMV infection during pregnancy					

Q8: Thinking back over the last 4 weeks of pregnancy, how often have you done each of the following activities?

Please tick the response that corresponds to you the most.

	Always	*Usually*	*Occasionally*	*Rarely*	*Never*
Wash my hands after changing a dirty (poo) nappy					
Wash hands after changing a wet nappy (urine only)					
Wash hands after wiping my child's nose					
Put my child's dummy in my mouth (for example, if fallen on floor)					
Eat left-overs on my child's plate					
Drink from my child's cup or bottle after they have had a drink from it					
Kiss my children on the lips					
Kiss children on the forehead					

Q9: Please rate how difficult it has been for you to do each of the following activities to reduce the risk of catching CMV

	Very easy	*Easy*	*Neither easy nor difficult*	*Difficult*	*Impossible*
Washing my hands after changing a nappy					
Washing my hands after wiping my child's nose					
Not putting my child's dummy in my mouth (for example, if fallen on the floor)					
Not eating left-overs from my child's plate					
Not drinking from my child's cup or bottle after they have had a drink from it					
Not kissing my child on the lips					

Q10: Did you watch the film about CMV from the study?

Yes ☐ No ☐

Q11: Have you looked for information about CMV from any sources?

Yes ☐ No ☐

If yes, where have you looked for information about CMV?

☐ Books
☐ Google search
☐ CMV action
☐ NHS Choices
☐ Asked Midwife/GP/Obstetrician
☐ Other (please specify)

Thank you, you have reached the end of the questions!
