# Supplementary material for: Changing knowledge, attitudes and behaviours towards cytomegalovirus in pregnancy through film-based antenatal education: a feasibility randomised controlled trial of a digital educational intervention
Source: BMC Pregnancy Childbirth. 2021 Aug 18;21:565. doi: 10.1186/s12884-021-03979-z (PMC8375137; doi:10.1186/s12884-021-03979-z)
Supplement: Supplementary file 7 — Additional file 7: Supplementary Figure S1. Graphs showing familiarity of participants with conditions affecting newborn infants. [file 12884_2021_3979_MOESM7_ESM.pdf]

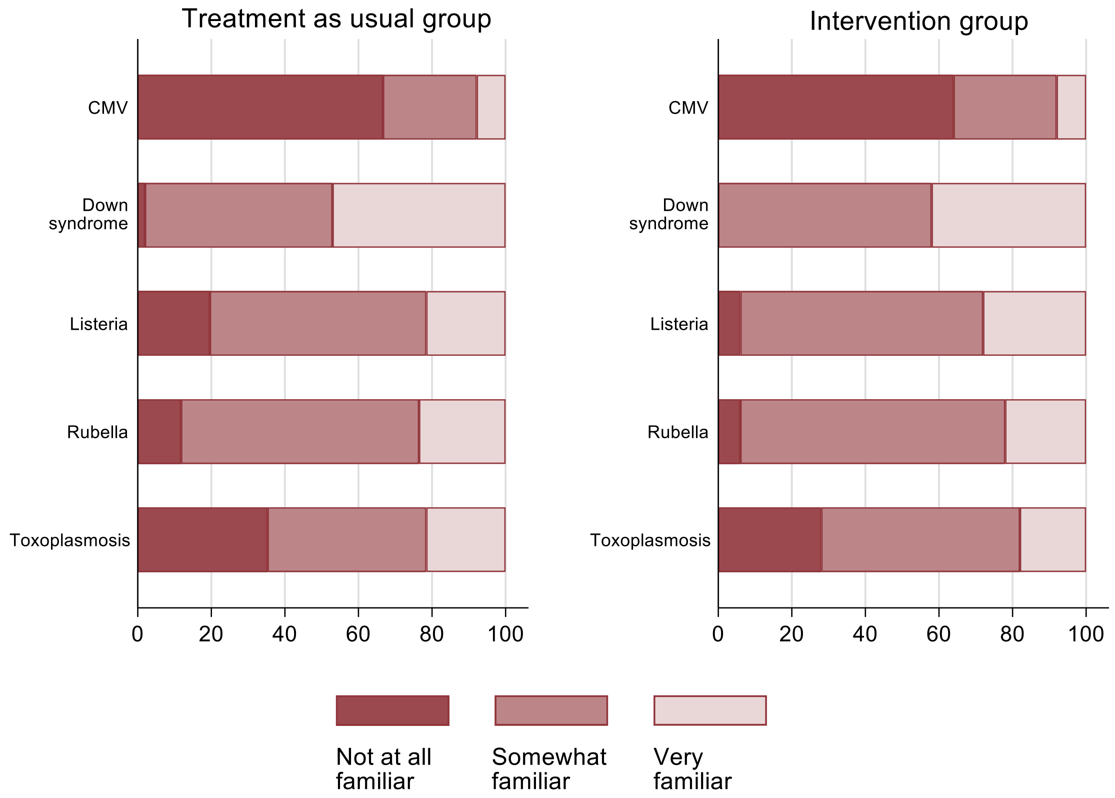

CMV Cytomegalovirus

Supplementary figure 1: Familiarity of participants with conditions affecting newborn infants
