# Supplementary material for: Changing knowledge, attitudes and behaviours towards cytomegalovirus in pregnancy through film-based antenatal education: a feasibility randomised controlled trial of a digital educational intervention
Source: BMC Pregnancy Childbirth. 2021 Aug 18;21:565. doi: 10.1186/s12884-021-03979-z (PMC8375137; doi:10.1186/s12884-021-03979-z)
Supplement: Supplementary file 8 — Additional file 8: Supplementary Table S1. Table of anxiety and depression scores for intervention and treatment as usual groups at baseline and 34 weeks. [file 12884_2021_3979_MOESM8_ESM.pdf]

Supplementary table 1: Anxiety and depression scores for intervention and treatment as usual groups at baseline and 34 weeks

		BASELINE			34 WEEKS			TAU	INT	BASE LINE	34 WEEKS	TAU	INT
		INT (n=51)	TAU (n-52)	BETWEEN GROUPS (PP)	INT (n=51)	TAU (n=52)	BETWEEN GROUPS (PP)	WITHIN GROUP (PP)	WITHIN GROUP (PP)	BETWEEN GROUPS (ITT)	BETWEEN GROUPS (ITT)	WITHIN GROUP (ITT)	WITHIN GROUP (ITT)
				BETWEEN			BETWEEN						
**Anxiety**	Mean (SD)	13.22(2.59)	13.06(3.17)	0.360	15.53(5.75)	14.11(3.49)	0.646	0.074	0.160	-	0.190	0.172	0.458
	Median (Q1-Q3)	13(12-15)	13(11-14)		13(12-17)	14(12-16)							
	Range	9-19	9-27		9-34	9-27							
	Missing	1(2%)	0(0%)		15(29.4%)	7(13.5%)							
**Depression**	Mean (SD)	3.47(2.77)	2.98(2.97)	0.227	5.81(5.29)	4.44(4.04)	0.216	**0.019**	**0.004**	-	0.390	0.087	**0.0002**
	Median(Q1-Q3)	3(1-5)	2.5 (0.5-4)		5(2.5-7)	4(1-6)							
	Range	0-13	0-12		0-27	0-18							
	Missing	0(0%)	0(0%)		15(29.4%)	7(13.5%)							

INT Intervention; TAU Treatment As Usual; PP Per Protocol; ITT Intention To Treat; SD Standard Deviation; The ITT analyses were conducted using permutation tests associated with continuous outcomes (paired for within group comparisons, unpaired for between group comparisons) under missing completely at random assumption for missing observations.
